# The tidal wetland restoration goldmine: Prospects and pitfalls of mining confined disposal facilities

**DOI:** 10.1017/cft.2026.10046

**Published:** 2026-07-21

**Authors:** Justin L. Shawler, Brian D. Harris, Brooke M. Walker, Susan E. Bailey, Paul Schroeder

**Affiliations:** 1 Coastal and Hydraulics Laboratory, US Army Engineer Research and Development Center, USA; 2 Interagency Airborne Technologies for Lidar, Analysis, and Surveying, USA; 3 Environmental Laboratory, US Army Engineer Research and Development Center, USA

**Keywords:** confined disposal facility, dredged material management area, beneficial use of dredged material, marsh restoration, wetland restoration, marsh nourishment

## Abstract

Saltmarshes along the United States South Atlantic Coast, and globally, face critical threats from sea-level rise, edge erosion, ponding and historical human modifications, all of which are exacerbated by sediment deficits. Uncontaminated sediment stored in confined disposal facilities (CDFs) represents a significant and often overlooked resource for marsh restoration. As a result of management practices in the second half of the twentieth century, many unnaturally high earthen structures occur adjacent to drowning saltmarshes in the United States. In this perspective article, we explore the potential for mining CDFs to provide the sediment necessary to restore and sustain at-risk saltmarshes. Using publicly available data from the US South Atlantic coast, we estimate that reclaiming sediment from existing CDFs could offset sea-level rise impacts by 2100 for 13% of South Atlantic marshes. While the potential benefit is substantial, scientific, planning and implementation hurdles are presented. These pitfalls include potential issues with sediment characterization, limited guidance on chemical evaluation to reduce environmental risks, construction complications due to soft soils and legal and financial considerations. Despite these barriers, we conclude with a path forward for researchers and practitioners to address the hurdles through dedicated case studies, adaptive management principles and stakeholder engagement.

## Graphical abstract

**Figure:**
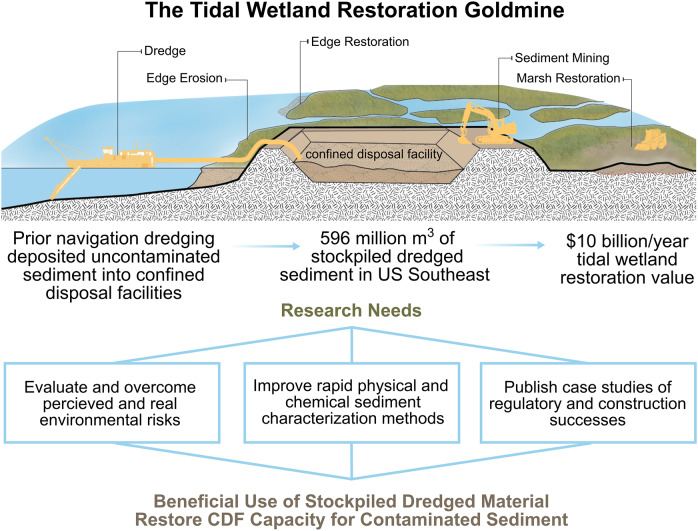

## Impact statement

This perspective moves beyond identifying the problem of wetland sediment deficits by providing a tangible solution. We demonstrate that reclaiming material from uncontaminated dredged material stockpiles called confined disposal facilities is not only feasible but essential for large-scale coastal restoration to better protect our coastal communities. By outlining a clear path forward that addresses key hurdles in research, planning and implementation, this article provides practitioners with the strategic framework needed to turn this concept into a cornerstone of sustainable coastal management.

## Key points

1. Over 596 million m^3^ of dredged material currently stockpiled is potentially available for restoration of US South Atlantic marshes.
2. Sediment reserves offer potential value of $10 billion annually to raise saltmarshes 1.1 m to address sea-level rise from 2000 to 2100.
3. Numerous challenges and hurdles must be overcome to successfully use these stockpiles for marsh restoration.

## Introduction

**Figure 1.**
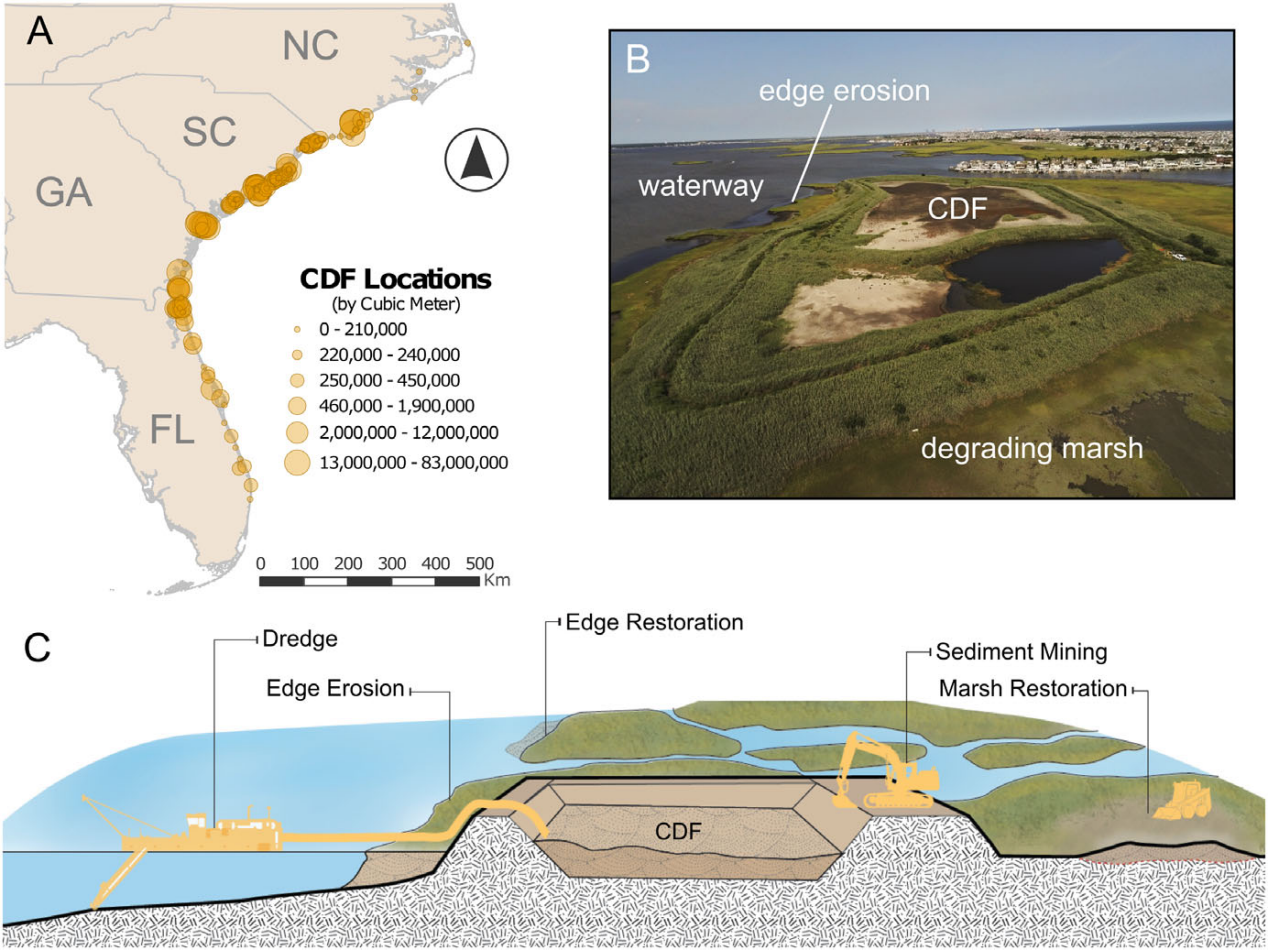
A) Confined disposal facilities (CDF) and volumes along the US South Atlantic coast. B) Example of a CDF located along a waterway and adjacent to a degrading marsh in coastal New Jersey, USA. Photo credit: David White/USACE. C) Conceptual diagram of CDF sediment management and beneficial re-use for saltmarsh restoration. Figure 1. long description.

The loss of 46.6% of global saltmarsh underscores the urgent need for innovative coastal restoration strategies (Brook et al., 2025; Suedel et al., 2021). Traditional sediment sourcing from inland and offshore borrow areas is becoming prohibitively expensive and potentially environmentally damaging (e.g., Nairn et al., 2004). While recent guidance in the United States promoting the beneficial use of dredged material (118th Congress, 2024) is a positive step, it does not fully address the saltmarsh sediment deficit. A more comprehensive solution may lie in an often-overlooked resource: the vast stores of sediment locked away in coastal confined disposal facilities (CDFs) (Figure 1).

CDFs are essentially stockpiles of dredged material that represent a sediment management solution following the passage of the Clean Water Act in 1972. CDFs are a necessary tool to maintain marine navigation. Although environmental testing would be required, most sediment in CDFs outside of highly industrialized, contaminated zones is likely suitable for coastal restoration. Many of these facilities are at or nearing capacity, which limits their future use and could compromise coastal navigation. Building new CDFs is often infeasible due to high construction and permitting costs (HDR and LMS, 2006). A more practical approach involves emptying existing CDFs and repurposing the sites as processing facilities for future sediment (Bailey et al., 2010), transforming them into a largely untapped “goldmine” for coastal restoration.

With this perspective piece, we aim to spur action by highlighting the strong potential of CDF sediment as a solution for coastal restoration. Using the southeast US Atlantic Intracoastal Waterway, a shallow-draft estuarine navigation channel located between offshore barrier islands and the mainland coast, as a case study, we quantify the available CDF sediment and its potential benefits to adjacent marsh environments. Next, we identify challenges to large-scale CDF mining and propose a research framework for sustainable CDF management. The insights and strategies presented here are broadly applicable to coastal regions worldwide, offering a path toward more resilient coastal environments.

## Coastal restoration needs

Coastal areas are among the most densely populated in the United States, with the southeast states experiencing particularly rapid growth, now exceeding 20 million residents (NOAA Office for Coastal Management, n.d.). These communities, along with seasonal tourists, depend heavily on the services of over 400,000 ha of saltmarsh along the US southeast coast (SASMI, 2023). These marshes act as vital natural infrastructure, reducing storm surge and flooding impacts (Wamsley et al., 2010; Möller et al., 2014; Narayan et al., 2017; Rezaie et al., 2020) and providing habitat for numerous species (Boesch and Turner, 1984; Rush et al., 2009). With an estimated ecosystem service value of $193,843 per hectare annually, the marshes of the US South Atlantic alone are worth ~$78 billion per year (Mitsch et al., 2015; SASMI, 2023).

Despite their immense value, these critical ecosystems are in decline due to a combination of threats, including sea-level rise, historical land claims, edge-erosion and anthropogenic disturbances (Day et al., 2011; Couvillion et al., 2017). While saltmarshes can naturally migrate inland to adapt to rising seas, this migration is often blocked by development and steep slopes (Enwright et al., 2016; Molino et al., 2022). This “coastal squeeze” amplifies the impacts of vegetation loss and pond expansion, which degrade marsh function (Mariotti, 2016; Cadigan et al., 2022).

To counter these losses, solutions that increase sediment supply, such as the beneficial use of dredged material, are essential for raising marsh elevation (Ganju, 2019; Suedel et al., 2021). Techniques like hydraulic pumping and nearshore berm placement are growing in practice (Harris et al., 2021; Piercy et al., 2023; Perkey et al., 2024; Harris et al., 2026). However, beneficial use of dredged material during navigation maintenance events alone cannot offset the scale of marsh degradation. Additional sediment sources are desperately needed, but conventional upland and offshore sand are often prioritized for beach nourishment projects and are typically considered too costly for large-scale marsh restoration. This is why we must turn to a largely overlooked resource lying adjacent to these threatened marshes: the sediment stored in CDFs.

## Prospects of the coastal restoration goldmine

While the beneficial use of dredged material is a critical first step toward restoring wetlands, annual dredging cycles cannot supply the sediment volume required to maintain marsh productivity against projected rising seas. The reclamation of material from CDFs, however, presents a transformative and sustainable solution. Wetland nourishment can utilize the full range of grain sizes found in CDFs, unlike other beneficial uses often limited to sand, such as beach nourishment.

The concept of reclaiming CDF sediment is not new, dating back to the 1970s with proposed uses like wetland creation, mine reclamation and landfill capping (Saucier, 1976). A series of studies in the early 2000s further developed frameworks for assessing and processing CDF material, aiming to establish a circular management system where sediment is only stored temporarily (Olin-Estes and Palermo, 2001; HDR and LMS, 2006; Bailey et al., 2010). Although limited, successful case studies—including beach nourishment, construction fill and island creation—demonstrate that removing and repurposing this material is feasible (Taylor Engineering, Inc., 2020).

This opportunityʼs scale is immense. Along the southeast US Atlantic Intracoastal Waterway alone, an estimated 596 million m^3^ of dredged material is stored in 164 CDFs (Figure 1a; USACE, 2024). This sediment has the potential to offset projected sea-level rise (Sweet et al., 2022) for 149,000 ha of saltmarsh (37% of US Southeast Atlantic marshes) until 2050 or 54,000 ha of saltmarsh (13% of US Southeast Atlantic marshes) until 2100. If used for wetland restoration (Mitsch et al., 2015), the total ecosystem service value of sediment stored in CDFs is at least $48/m^3^ per year ($62/yd^3^ per year) and represents a total potential saltmarsh restoration value of $10 billion per year based on raising saltmarshes 1.1 m to address sea-level rise from 2000 to 2100. Even accounting for the need to iteratively place sediment to stay within the local tidal range, this represents an enormous potential value. Tapping into this resource is a compelling prospect, but significant planning and implementation hurdles must be overcome to make large-scale CDF mining a widespread reality for coastal restoration.

## Pitfalls of the coastal restoration goldmine

While CDFs represent a potential “goldmine” for coastal restoration, mining this resource is fraught with significant pitfalls that span planning, environmental and operational domains. Like many ambitious restoration projects, these efforts can stall in the planning phase if the complexities are not proactively addressed (e.g., Abelson et al., 2020).

### Sediment characterization and feasibility

The first hurdle is sufficient site assessment. A project is only viable if the CDF contains sufficient sediment volume of the right type to justify the high costs of sediment extraction and relocation. However, CDFs are not uniform stockpiles. Their complex histories of receiving mixed sediments, which are often reworked for berm maintenance or dewatering, create highly heterogeneous pockets. This makes it difficult to accurately quantify the percentages of sand and fines, a critical step that dictates both the ideal beneficial use (marsh, beach or upland; Saucier, 1976; Bailey et al., 2010) and the necessary extraction equipment (Olin-Estes and Palermo, 2001; Olin-Estes et al., 2002).

### Environmental risks

A primary concern for CDF sediment extraction for wetland restoration is contaminant risk. While material is often tested before being placed in a CDF, the standards for confined disposal are different from those for unconfined use in saltmarshes (Bailey et al., 2010). Contaminant mobility can change significantly within a CDF as aerobic processes alter sediment chemistry, meaning that original pre-dredge testing required by the Clean Water Act (Schroeder et al., 2006; Schroeder et al., 2008) is often insufficient (Hoeppel et al., 1978). Furthermore, large-scale placement of mixed-grain-size material could have unknown cumulative impacts on marsh biodiversity and sediment transport as colonization patterns are tightly linked to sediment properties and elevation (Stoorvogel et al., 2024; Wilburn et al., 2024). While contamination in industrialized areas and their CDFs (e.g., Indiana Harbor and Canal; Ingersoll et al., 2002) is usually well documented, material such as that found along much of the southeast US Intracoastal Waterway is not typically associated with a long industrial history where the presence of industrial contaminants is as common. As such, these sites are likely more viable and warrant detailed sampling and analysis to verify their viability. Currently, optimized guidance for characterizing and sampling CDFs for beneficial use is still lacking.

### Logistical and construction risks

Coastal environments present inherent challenges, including extremely soft soils, tidal fluctuations and sensitive wildlife habitats that restrict access and working windows (Jafari et al., 2019; Harris et al., 2025). The structural integrity of the CDF itself is a concern as equipment must operate on potentially unconsolidated material and steep containment slopes. Transportation methods, often the costliest component of the project, must be carefully selected to enable equipment mobilization and use over soft soils and minimize impacts on surrounding habitats. The presence of hidden debris from past dredging and construction (e.g., wood scabbing and pipeline offcuts; Bailey et al., 2010) can interfere with extraction machinery, causing costly delays. As with beneficial use of dredged material, adding material to wetland and subtidal surfaces leads to compaction of both the placed and underlying materials. The result is that there is not a 1:1 ratio of elevation gained to sediment placed.

### Legal and financial hurdles

Overarching these technical challenges are significant administrative hurdles. Securing adequate funding is a primary obstacle. Furthermore, projects often involve multiple land parcels for extraction, transport and placement, creating a complex web of legal and liability concerns. Establishing clear ownership of the sediment itself and the authority to transfer it can also be a surprising roadblock requiring extensive time to navigate permits and agreements.

### The need for adaptive management

Given that CDF reclamation is a nonroutine construction activity, many challenges are unforeseeable. This underscores the critical need for adaptive management. Continuous monitoring during and after construction is essential to ensure project objectives – such as achieving target marsh elevations, minimizing impacts on existing habitats and evaluating the need for reapplication of dredged material – are met, allowing teams to adjust their approach in real time (Harris et al., 2026).

## The path forward

Forging a path forward requires a multifaceted approach that addresses challenges in research, planning and implementation. By systematically tackling these hurdles, the immense potential of CDFs can be unlocked for large-scale coastal restoration.

### Ecological and sediment implications

The initial challenges are stacked toward research, particularly understanding the potential impacts of variable grain sizes on wetland biodiversity and sediment transport. While thicker sediment placements can cause initial vegetation dieback (Walters and Kirwan, 2016; Raposa et al., 2022), studies indicate these effects are often temporary and ultimately lead to greater stability in the long-term. For the placement of dredged material in wetlands, research shows native vegetation recovery within three to four growing seasons regardless of dredged material thickness (Raposa et al., 2022; Raposa et al., 2023; Harris et al., 2025), almost immediate use by avian species (Harris et al., 2026) and multiyear recovery of infaunal communities (Posey et al., 1997; Fleeger et al., 2020). Moreover, studies point to increased plant productivity (VanZomeren et al., 2018) and significant long-term biodiversity gains from beneficial use projects (Suedel et al., 2021). The key insight is that marsh function is controlled by a complex interplay of elevation, soil nutrients and microbial communities, not just grain size alone (Stagg and Mendelssohn, 2010; Jafari et al., 2024; Liu et al., 2024). Additionally, knowing the heterogeneity of the grain size in the saltmarsh of interest would inform the importance of knowing the consistency (or lack thereof) of the grain size of the dredged material. Future research should therefore focus on how to leverage the natural sorting of mixed-grain material, which creates gradients of elevation and soil type, to strategically enhance habitat diversity for species like shorebirds and terrapins.

### Planning and collaboration

Beyond research, the path forward must address significant planning-level concerns spanning stakeholder engagement, regulatory frameworks and technical requirements. For stakeholder engagement, planners can draw from successful community-informed approaches implemented in coastal Louisiana (Hemmerling et al., 2020). A key advantage is that CDF reclamation projects often achieve stakeholder buy-in more easily than traditional dredging; stockpiled material decouples restoration schedules from rigid navigation timelines, offering greater operational flexibility. This process can be further streamlined by sediment-match portals, such as Maryland’s “Beneficial Use: Identifying Locations for Dredge” tool (Specht, 2019), which align the interests of sediment “brokers” (such as the US Army Corps of Engineers) with coastal restoration practitioners. These tools also help to identify and justify projects in sediment-starved marshes using remote sensing metrics like the unvegetated-to-vegetated ratio (Ganju, 2019).

To navigate regulatory and contaminant testing hurdles, the path forward should leverage lessons from successful wetland beneficial use of dredged material projects. New Jersey’s Seven Mile Island Innovation Lab (SMIIL), for example, reduced stakeholder concerns through pilot projects and open dialogue, demonstrating a highly collaborative, science-based approach founded on “trust, persistence, [and] proactive leadership” among diverse partners (Chasten et al., 2023). For contaminant testing, planners must account for potential mismatches between current sediment chemical quality of CDF material and requirements for use in wetland restoration, which may vary from state to state in the United States. In such cases, it may not be cost-effective or feasible to treat the sediment.

Practitioners can overcome physical characterization hurdles by utilizing rapid assessment methods during the planning phase. Technologies like ground-penetrating radar can characterize the distribution of sand and mud, while dynamic cone penetrometer tests assess soil strength (Gallegos et al., 2026). These methods allow for more targeted use of costly physical sampling to confirm material chemical suitability (Estes et al., 2012). However, for these rapid characterization techniques to be widely adopted, dedicated studies must continue to demonstrate their specific successes and limitations.

Finally, project teams can leverage lessons learned from beneficial use of dredged material projects to predict how soil consolidation will impact the success of using CDF sediment for wetland restoration. Numerous recent papers detail the geotechnical methods and modeling required to understand wetland consolidation (e.g., Harris et al., 2025; Zoccarato et al., 2025). For example, consolidation and surface erosion reduced original placement thicknesses by 60% at a site in coastal New Jersey (Harris et al., 2025). Developing these sorts of estimates using current best practices can increase the likelihood of project success.

### Funding and implementation

In terms of implementation, funding and construction methods remain the biggest challenges. Construction methods can be tested by similarly leveraging lessons learned from SMIIL, which involved the use of early pilot projects before taking a more system-wide approach (Chasten et al., 2023). Additionally, implementation success will require early (pre-bid) engagement (e.g., Mehany et al., 2018) with marine contractors and construction experts who have experience with soft sediment removal and transportation options specific to marshes. Thankfully, sediment transportation costs, which are high for many beneficial uses of CDF sediment opportunities (HDR and LMS, 2006), should be relatively low as sediment could be directly placed or fluidized and pumped only a short distance given the proximity of most CDFs and surrounding marshes (Figure 1b, c). While funding could be the biggest hurdle, cost-sharing may split expenses and associated risks among stakeholders (federal, state, private and nonprofit conservation landowners) who value saving existing wetlands and waterway managers who need to free up capacity in CDFs. Funding for pilot projects could leverage National Fish and Wildlife Foundation funding and similar conservation grants aimed at moving projects through conceptualization, design and implementation phases.

## Summary

Many coastal saltmarshes are degrading under a critical sediment deficit. This perspective proposes a practical but overlooked solution: reclaiming the vast dredged sediment “goldmine” stockpiled within CDFs. Using this material restores vital ecosystems while freeing additional capacity in CDFs for sediment unsuitable for restoration. While significant research, planning and operational hurdles exist, they are surmountable. We have outlined a strategic path for researchers and practitioners to overcome these challenges, turning a legacy liability into a key component of coastal resilience.

## Data Availability

The data referenced in text and included in Figure 1a are available from USACE (2024) and further summarized by Taylor Engineering, Inc. (2020).

## Figure 1. Long description

(a) Map of Confined Disposal Facility (CDF) locations and volumes along the U.S. southeast coast showing orange dots for each CDF. There are concentrations of high volume CDFs along the South Carolina coast in particular, (b) Low angle aerial photo of a CDF with earthen dikes next to a degrading marsh as indicated by ponding and edge erosion, and (c) Diagram of a dredge pumping material into a CDF (which was common practice in the late 1900s) and excavators removing material to place on a marsh for restoration (which is the solution proposed by this perspective piece).

Navigate back to Figure 1..
