# Supplementary material for: The tidal wetland restoration goldmine: Prospects and pitfalls of mining confined disposal facilities
Source: Camb Prism Coast Futur. 2026 Jul 21;4:e22. doi: 10.1017/cft.2026.10046 (PMC13458092; doi:10.1017/cft.2026.10046)
Supplement: Peer review history

# Reviewer report: Review: The tidal wetland restoration goldmine: Prospects and pitfalls of mining confined disposal facilities — R0/PR2

**DOI:** 10.1017/cft.2026.10046.pr2

**Authors:** Thomas McGinnis

**Affiliations:** United States

Mining Confined Disposal Facilities (CDFs) to address marsh surface elevation and sediment limitations is relevant, and a perspective article from USACE employees is a good introduction to the issues involved with using CDFs. Three issues to address are:

(1) Elaborate a little more on the work that has been done in Louisiana towards this issue. Programs such as CWPPRA has utilized BUDMAT and has attempted to mine CDF, especially along the Calcasieu Ship Channel. Mining CDFs has proven to be tricky. This may strengthen your perspective.

(2) Develop the premise described at the end of Sect 3 regarding raising saltmarshes 0.4 m to address sea-level rise from 2000 to 2050.

a. Sediment additions are typically within the context of remaining within the tidal prism which may require an iterative approach over years.

b. If possible, update the date range from 2000 to 2050.

(3) Ecological and Sediment Implication: knowing the heterogeneity of the grain size in the saltmarsh of interest would also inform the importance of knowing the consistency (or lack thereof) of grain size of the dredged material.

Otherwise, my remaining comments are editorial in nature:

39 and 46: Use Oxford Comma in complex sentences and compound lists. This was done well elsewhere in the ms.

66: Use “goldmine” (figurative/conceptual) rather than “gold mine” (actual, specific location). “Goldmine” was used throughout the rest of the ms.

80-81: Combine paragraphs

107: Don’t split technical terminology/lingo with “annually”. Try this: While the beneficial use of dredged material....wetlands, annual dredging cycles cannot...

110-111: Change the order and add an example: Wetland nourishment...unlike other beneficial...sand such as beach fill.

158 and 177: Remove comma

219: Add “(SMIIL)” for later usage in Line 232.

241: Remove commas after “expenses” and “risks”

241: Add “stakeholders”: ...risk among stakeholders (federal, ...landowners)

243: Delete “specifically”.

References Section (check throughout for these examples)

- “and” and “&” are both used in authors' lists; pick one for consistency.

309: All caps for journal title

312: Authors' list format different from others.

# Reviewer report: Review: The tidal wetland restoration goldmine: Prospects and pitfalls of mining confined disposal facilities — R0/PR3

**DOI:** 10.1017/cft.2026.10046.pr3

This perspective manuscript outlines arguments for the reuse of sediment stored in CFD for environmental restoration projects. The authors highlight the value of this stored sediment, then possible barriers and finally suggest solutions based mainly on research, funding, and early planning. This is a timely perspective suggesting a single solution to solve two problems: reuse of unused sediment kept in CDFs to feed retrieving saltmarshes.

I have only a few minor comments to the perspective.

I would urge the authors to include information also about the barriers and paths forward in the abstract. As it reads now, the abstract has a long introduction to the subject, but it does not represent the manuscript.

Within the environmental risks section, it isn’t clear how much of the confined stored sediment is expected to actually be clean to the necessary standards/requirements in terms of hazardous chemicals. I am from Europe where there are requirements, so I am unaware of similar requirements in US. Assuming there are, it is important to put into perspective how clean the sediment kept in CDFs is, in relation to the levels they are expected to be for reuse in such projects.

It also misses some information on how to overcome potential mismatch between current sediment chemical quality of stored sediment and the requirements for use in wetland restoration projects. If the sediment is not clean to the necessary requirements, would it be feasible, both in practical terms and economically, to clean the sediment? I understand that this is a perspective, and thus not an exhaustive list, but perhaps mention this barrier that can be decisive for these projects.

Given that the manuscript is a perspective, and not a review, I would urge the authors to reduce the list of references substantially.

# Editor report: Recommendation: The tidal wetland restoration goldmine: Prospects and pitfalls of mining confined disposal facilities — R0/PR4

**DOI:** 10.1017/cft.2026.10046.pr4

**Affiliations:** Louisiana Universities Marine Consortium, United States

Associate Editor (Krauss): I think this perspective could make a nice contribution to Coastal Futures. I was unaware of this source of sediment, and I commend the authors for writing this perspectives article. Reviewers were in agreement, but also offered a number of edits to consider, particularly associated with contamination concerns (Reviewer 2). Also, the reviewers were split on requesting more relevant citations versus already having too many for this perspectives piece. I recommend being very diligent to include only the most important references throughout, perhaps selecting peer reviewed journal articles over government reports where you can. Also, delete citations to meeting talks. Those are not peer reviewed and are thus not scientifically authoritative.

Major point:

There are important reasons why we do not implement BUDM projects everywhere. Issues associated with all BUDM projects still apply to the usage of confined disposal facility usage, and should be mentioned. For example, adding new material to existing wetland surfaces (or even subtidal surfaces) lead to greater compaction, so it is not a 1:1 replacement. This will affect the overall volume of this sediment source in terms of potential area of benefit, and dampen its benefit for offsetting sea-level rise in any specific location. Also, BUDM projects often do need re-application plans, particularly when root zones do not establish rapidly. This swells costs significantly. Please interject these important details.

Minor points:

Abstract. Clarify what the 2100 and 13% refer to? Assuming 13% by 2100 in the modeling. Suggest stating that if so. 

Title does not really describe the story being told. Should add some reference to “for tidal wetland restoration”? How about something like “Opportunities, prospects and pitfalls of mining confined disposal facilities for tidal wetland restoration”?

For the use of the phrase “U.S. Southeast Intracoastal Waterway”, consider defining for the reader what this is? The US is unique in having this infrastructure, so it might not be too clear for international readers.

I look forward to reading your revised manuscript. Please detail how all comments were addressed in your cover letter or appropriate space.

# Editor report: Decision: The tidal wetland restoration goldmine: Prospects and pitfalls of mining confined disposal facilities — R0/PR5

**DOI:** 10.1017/cft.2026.10046.pr5

**Affiliations:** Geography, Cambridge University, United Kingdom of Great Britain and Northern Ireland

No accompanying comment.

# Editor report: Recommendation: The tidal wetland restoration goldmine: Prospects and pitfalls of mining confined disposal facilities — R1/PR7

**DOI:** 10.1017/cft.2026.10046.pr7

**Affiliations:** Louisiana Universities Marine Consortium, United States

Associate Editor (Krauss): Very nice job with the revisions! I like this paper a lot and I feel that it will be quite useful. People talk a lot about nature-based solutions, but this has actual potential and is a straightforward approach, though expensive. I have a couple of additional edits to suggest, but this is ready to be accepted after you consider these. No need for a reconciliation. My first comment is my biggest concern for you all! Nice job on this Perspective.

L. 69. Do the authors intend to suggest using CDFs as a “primary” tool, or as a tool? Primary meaning prioritize this over all other restoration approaches (e.g., FW diversions, dredged sediments, gapping, terracing)? Just checking. Worried that Corps leadership might not want this stated in this fashion.

L. 82. Space after “)”

L. 167. Suggest deleting this sentence: “Beyond planning, operational logistics can be a nightmare.” Use of “nightmare” is strange, and turns out to be redundant with next sentence.

L. 222. “a priori” is not used properly. It cannot be used to split an infinitive. I would take a priori out. It’s implied by the rest of the sentence.

# Editor report: Decision: The tidal wetland restoration goldmine: Prospects and pitfalls of mining confined disposal facilities — R1/PR8

**DOI:** 10.1017/cft.2026.10046.pr8

**Affiliations:** Geography, Cambridge University, United Kingdom of Great Britain and Northern Ireland

No accompanying comment.
